# Video game training in traumatic brain injury patients: an exploratory case report study using eye tracking

**DOI:** 10.16910/jemr.15.1.6

**Published:** 2022-06-15

**Authors:** Elham Azizi, Joanne Fielding, Larry A. Abel

**Affiliations:** Mashhad University of Medical Sciences, Mashhad, Iran; Monash University, Melbourne, Australia; University of Melbourne, Melbourne, Australia

**Keywords:** Eye movement, eye tracking, saccades, microsaccades, antisaccades, smooth pursuit, scanpath, convergence, attention

## Abstract

Remediation of attentional impairments is an essential component of cognitive rehabilitation after traumatic brain injury (TBI). Evidence from healthy participants has demonstrated attentional improvement following playing an action video game. This exploratory study investigated its application in TBI participants in a multiple baselines single case experimental design (SCED). Saccadic eye movements, recognized as the visible indicators of visual attention, were assessed to evaluate the effectiveness of the game training. Three severe TBI participants were trained in an action game for 10 hours. Saccadic eye movements during a self-paced saccade and an abstract visual search task were investigated during baseline, mid training and post-training. Using Percentage of Non-overlapping Data (PND), analysis showed consistent increase in the rate of the self-paced saccades in participants 1 (PND=80%) and 2 (PND=70%). In abstract search, fixation duration showed a minimally effective decrease for participant 2 (PND= 60%) and a moderately effective reduction in participant 3 (PND= 80%). Search time showed a highly effective reduction in participant 2 (PND = 100%) and moderately effective decrease in participant 3 (PND=70%). Overall, video game training might modify allocation of attention in eye movements. More evidence is required to validate the usefulness of this novel method of the cognitive training.

## Introduction

Attentional impairments are among the most pervasive deficits following traumatic brain injury (TBI). At any one time, we are exposed to far more sensory information than the brain can process. Attention allows us to actively select and enhance elements of this information that are important to us, and are required for further processing from that which is irrelevant and can be filtered out (35). TBI patients may suffer from mental slowness (68) or report problems in performing more than one task at a time (8; 75). They may distract easily over time or having problems in planning or switching between tasks (52; 67). This suggests attention as a multidimensional phenomenon which can be divided into specific aspects (58).

For more than a decade, action video game playing has been used as a novel technique to improve various aspects of attention in healthy young adults (16; 21, 22; 50; 56; 77) and in groups such as the elderly (4; 6), people with stroke (46), children with dyslexia (20) and amblyopia (36; 48). TBI patients may benefit similarly.

According to the American Congress of Rehabilitation Medicine on Brain Injury, remediation of attention problems should take place during the post-acute period following a TBI, and should include both direct attention training and meta-cognitive training to promote recovery of damaged neural circuits, boost compensatory strategies and facilitate transfer to everyday life tasks (11; 12). While direct attention training explicitly addresses attentional impairments through a range of graded exercises (65), meta-cognitive training involves self-monitoring through performance feedback and encouragement of effort and motivation (11).

During action game playing, one may practice various visual attention tasks simultaneously, including fast monitoring of all the display for upcoming events, multiple objects tracking and distractor rejection. As a trainee progresses through the game, the level of game difficulty increases, and a variety of new tasks are encountered. In short, the stimuli that a trainee encounters might be considered as graded exercises, consistent with direct attention training. Moreover, at any level feedback is provided to the player by showing the scores that the player has obtained, the number of lives, deaths and the gun supplies. In some cases, when the player reaches a certain score, extra scores are given as a bonus, possibly increasing the player’s motivation and desire to continue play. Progressing through the game gradually would involve the player in the game, leading to increase in arousal and motivation, both known to facilitate the process of learning and acquiring new skills (24; 38; 62). This may act as a way similar to the metacognitive training (11). Playing an action video game as a rehabilitative strategy is more engaging and appealing for trainees than current methods of cognitive training based on perceptual learning with typically simple, repetitive and boring tasks with low compliance rate (2).

To explore the potential of action video game playing as a therapeutic tool, Vakili and Langdon (70) examined performance in two TBI groups. The experimental group participated in 2 hour training sessions over 8 weeks (90 minutes of playing the game “Medal of Honor” and 30 minutes to do a psychoeducational program). The control group received no intervention. Participants were tested on the attentional blink task (AB) (63) and the Test of Everyday Attention (TEA) (59). The AB task is commonly used in evaluating visuo-temporal processing of information and the TEA comprises 8 subsets which assesses three aspects of attentional functioning including selective attention, sustained attention and mental shifting. An enhancement in game performance and in AB (reduction in AB in all time lags) from pre- to post-training in the action video game training group was revealed, while the control group did not show a change. In TEA, the experimental group showed enhancement in three categories of the test; however, it was accompanied with a deterioration on control group performance, unlike expected. Nevertheless, they found a positive correlation between game performance (fewer deaths) and attentional outcome measure in TEA (elevator counting with distraction) in the experimental group. Taking into account the limitations of the study, including having no active control group, and having two modes of training, authors proposed action video game training as an economically viable option for brain injury rehabilitation units.

To further explore the influence of action video game training on attention, this exploratory study examined its effectiveness by assessing eye movements using a simple self-paced saccade task (55) and a visual search paradigm (5). One of the fundamental functions of visual attention is selecting regions that contain useful information for programming of saccadic eye movements to land on that region (29; 39; 71). A shift in the line of sight (i.e. a saccade) represents an overt shift of attention. Participants show better perceptual identification of the target at the saccade goal than elsewhere and when attention is allocated towards a target, saccade reaction times are shorter towards that target (29; 39). Directly measuring saccadic eye movements enables us to monitor shifts of attention.

Saccadic eye movements are generated and controlled by areas in the frontal lobe (such as prefrontal cortex, frontal eye field and supplementary motor area), superior colliculus, pons and medulla (53; 66). Focal lesions in the frontal lobe commonly occurs after a TBI (8; 47). Diffuse brain damage also occurs throughout subcortical white matter, particularly in the corpus callosum and brainstem, over the course of hours or days after the injury (64). Perhaps not surprisingly, saccadic eye movements are often impaired following a TBI (26; 27; 74). Studies have found deficits in the generation of volitional saccades. For patients with various severities of TBI, these deficits include prolonged antisaccade latencies, higher error rates for antisaccades and memory guided saccades, poorer accuracy for antisaccades (25; 42), and fewer self-paced saccades within a limited time frame (74). Studies have demonstrated the potential utility of self-paced saccade task in the diagnosis of different severities of TBI and in post-concussion syndrome (34; 69). Decreased number of self-paced saccades was associated with reduced white matter integrity and higher symptom burden following post-concussion syndrome (69). Hunfalvay et al. (34) demonstrated a sensitivity of 0.77 and specificity of 0.78 of self-paced saccades in the diagnosis of different severities of TBI. They even proposed it as a test to monitor the rehabilitation progress following TBI.

### Present study

A multiple baselines single case experimental design (SCED) was used to evaluate the effectiveness of action video game training on improving visual attention examined by assessing eye movements in three severe TBI patients. SCEDs play a significant role in clinical research, especially in rehabilitative studies (40; 41; 60). In this design, subject’s behavior is repeatedly measured in absence and presence of an intervention, so each participant act as their own controls. Moreover, any intervention effect can be detected even via the large variability of the subject due to pain or fatigue, which is not considered in group design studies (40). SCEDs are especially useful in populations such as TBI (72) with patients differing in severity, site of injury, time since injury and level of functioning.

The tasks used were self-paced saccades and abstract conjunctive visual search. Self-paced saccade task contains voluntarily constant moving the eyes from one target to the other over a fixed period of time (1). Voluntary saccades are part of continuous attentional programming and attentional rhythm controls making or not making voluntary motor behavior (30). A pathway from the frontal eye field (FEF) and the dorsolateral prefrontal cortex (DLPFC) to the superior colliculus is crucially involved in generating self-paced saccades (49; 57), although a role for the anterior cingulate cortex in sustaining motivation for performing self-paced saccades has also been reported (69). If gaming is accompanied by an improvement in the shifting of attention, the rate of self-paced saccades should increase post training.

Visual search is a popular method for studying allocation and efficiency of visual attention; however, it can also investigate the performance of a subject and the intervention effect in more real situations. If one can search faster for a letter among distractors, it might generalize to searching for a brand of tuna in a super market shelves or search a friend in a crowd to name as examples. In visual search task, two variables were assessed: mean search time and mean fixation duration. Visual information is acquired during periods of fixation due to reduced sensitivity during a saccade (28). If gaming is accompanied by an improvement in the ability to efficiently resolve visual information i.e. enhanced information processing, duration of fixations and search time should reduce post training (43).

## Methods

### Participants

Three patients with a history of severe TBI were recruited through advertisements on the Brain Injury Australia Facebook page and the University of Melbourne online student notices. Volunteers were interviewed using the Ohio State University TBI interview form to determine whether or not there had been a TBI and then to determine severity of the injury (13). Inclusion criteria were: (a) age between 15-50 years, (b) history of severe TBI (Glascow coma scale, GCS < 9, Loss of consciousness, LOC>24 hours), (c) at least 6 months post injury, (d) no history of video game playing for at least one year prior to participation, (e) no history of any ocular motor or neurological condition before the injury, (f) intact motor abilities in both hands, enabling them to play a fast paced video game on a laptop using a keyboard and mouse.

### Patient 1:

Patient 1 was a 22-year old male involved in a motor vehicle accident 8 months prior to participation. He sustained a severe TBI [initial GCS score 3, increased to 7 in the emergency department, loss of consciousness (LOC) 10 days, post-traumatic amnesia (PTA) 1 month]. A Computed Tomography (CT) scan showed subdural hematoma, haemorrhagic contusions in the right frontal and left temporal lobes, a significant right to left midline shift and mild hydrocephalus. Prior to participation in this study he had completed rehabilitation for mobility and personal activities of daily living.

### Patient 2:

Patient 2 was a 45-year old female involved in a motor vehicle accident 10 years prior to participation. She sustained a severe TBI (GCS score 4, LOC 2 months, PTA 2 months), fractured pelvis, fractured right tibia and fibula and a dislocated left knee. She spent several weeks in intensive care and was subsequently transferred to a rehabilitation center, where she spent several months to improve her cognitive and physical functioning. At the time of participating in this study, her ongoing problems included discomfort in her legs, slowed thought processing and poor balance and co-ordination affecting her gait, which was slow, wide-based and generally unsteady. She was living with her father who assisted her in all aspects of her daily life. As her accident occurred overseas, medical reports were not available, but she reported damage primarily involved frontal and temporal lobe regions.

### Patient 3:

Patient 3 was a 19-year old female with a complicated history. Her TBI was not diagnosed at the time of her first hospitalization at age 14. According to her mother, she had vomited and had a severe occipital headache before falling asleep. She was unarousable in the morning and was taken to the hospital. A CT scan showed posterior fossa hematoma. She underwent occipital craniotomy with evacuation of a fourth intraventricular hemorrhage and inter-parenchymal hemorrhage. She developed hydrocephalus post-operation which required an external ventricular drain. She was unconscious for 3 days following the craniotomy. It took several days until she was able to remember the incident resulting in her TBI and communicated that she had received a hard blow with a soccer ball to the back of her head after school. At the time she felt dizzy. After she became medically stable, she began speech therapy. She also complained of diplopia and was given an eye patch. After all vascular investigations were negative; she was referred for comprehensive rehabilitation for mobility, activities of daily living, balance and vision. She also reported a second head trauma 4 years after the first accident. At this time, she suffered a blow to the head as a result of falling downstairs. She had headache, double vision and ataxia following this incident. Her medical report recorded a GCS score of 15 and no other systemic abnormalities. A CT scan and magnetic resonance imaging (MRI) revealed stable postoperative changes from occipital craniotomy associated with cerebral encephalomalacia and gliosis. At the time of participation in this study, she was attending University and living independently in dormitory accommodation.

## Apparatus and stimuli

### Self-paced saccades

Self-paced saccades were recorded using a SensoMotoric Instruments (SMI) monocular infrared video-based eye tracker (SensoMotoric Instruments, Berlin, Germany), that recorded left eye at 200 Hz with a spatial resolution of less than 1°. Stimuli were presented on a 20 inch LCD monitor (SAMSUNG, 2043BW, with a refresh rate 60 Hz and resolution of 1050×680 pixels) with patient sitting 67 cm from it with head stabilized using a chinrest. A 5-point calibration was conducted before recording eye movements and if the x-coordinate spatial accuracy was < 1°, eye movement recording (left eye) began. A fixation cross was presented in the center of the monitor. While recording, the fixation cross was substituted with two 1º black dots at 10º to the left and right side of the center for 30 seconds. Patient was asked to make volitional saccades between the two dots as fast and accurately as possible. The number of primary saccades was counted.

### Abstract conjunctive visual search taski

**Figure 1:**
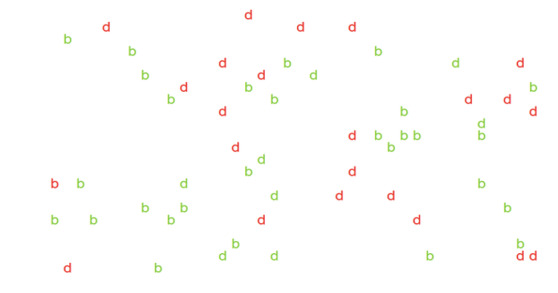
A trial of abstract conjunctive search. Participant’s task was to find a red letter “b” among distractors.

Eye movements throughout the abstract conjunctive search (76) were recorded using an Eyelink II head-mounted eye tracker (SR Research). Viewing was binocular, but the left eye only was tracked as there was no disconjucacy of the eyes in any subject. Eye position was sampled at 500 Hz with spatial accuracy of 0.5°. The search task was presented using a short-throw projector (NEC, WT610) on a screen at a viewing distance of 130 cm, resolution of 1024 × 768 and subtended 62.8° × 38.2° of visual angle on the screen. Task presentation and eye tracking were controlled with SR Research Experiment Builder software. The task trials were created by R statistical software (A language and environment for statistical computing, R Foundation for Statistical Computing, Vienna, Austria, http://www.R-project.org). Each trial contained 59 red and green letter “d”s with green “b”s as distractors and one red letter “b” as the target, randomly positioned on a white background (Figure 1). Each letter subtended approximately 0.6 °× 1° degrees of visual angle.

A 9-point calibration and validation were performed before starting the task and calibration was repeated if the spatial accuracy fell above 0.5°. Patient was required to locate a red letter “b” and press the button box as fast and accurately as possible. Each trial stayed on the screen until the subject reached his/her decision, indicated by pressing a button on a gamepad to end the trial or until an automatic time-out of 20 seconds. Ten trials were shown to the participant in every day of testing and training.

### Procedure

Eligible participants read the study information sheet and signed the consent form. They were reimbursed $10 per session for their participation as well as their public travel costs and parking fees. The study was approved by the University of Melbourne Human Research Ethics Committee (approval number: 1238522.1). Participants had normal or corrected-to-normal visual acuity as tested with a LogMAR chart at 3 meters and normal color vision as tested by the Ishihara test. Each participant was tested five times on five consecutive days on the self-paced and abstract search task to record baseline data. They subsequently began training, using the game Call of Duty, Modern Warfare II (Infinity Ward) for a total of 10 hours (1 hour per day for ten days), over a two-week period. Before each training session, participants were tested on the saccade and the abstract search tasks. Each session took 1 hour and 20 minutes to complete. Participants were also tested on the saccade and the abstract search tasks on two other occasions during the training period, once after 5 hours of training and once after finishing the training. Overall, the study included at least 17 sessions: 5 baseline testing sessions, 10 testing plus training sessions, 1 mid-training testing session, and 1 post-training testing session). These took at least 4 weeks for each participant to complete and did not include weekends.

### Testing procedure

On the first day of testing, tasks were explained to the patient and the experimenter ensured that the patient had learned the task by performing practice trials (five in each test) before recording. The testing procedure commenced with the self-paced saccade task. Following a variable rest period, the visual search task was presented to the patient.

### Training procedure

Game training was performed using a 15.5-inch laptop (Dell, Latitude E6520) with 1920 × 1080 resolution. The monitor subtended 35.3° × 19.6° visual angle, when the participant sat 55 cm from the laptop. In the first training session, the content and aim of the game, different movements and weapons were explained to the trainee, who started playing the game by completing the first level or ‘mission’, which served as a tutorial. The participants subsequently played the game for 1 hour, taking breaks when required. The experimenter sat in the testing room and was available to assist and give feedback if needed. Participants played the single-player mode of the game, which comprised 17 missions. If a participant finished the single player mode, they played the advanced Special Ops section which consisted of five new levels with new objectives. After finishing the daily training, the participant’s achieved level (mission) was recorded. If a mission was not completed, the participant started from that uncompleted mission the following day.

### Analysis

To investigate the influence of training on self-paced saccade rate and duration of fixation and visual search time in abstract search task, we used a procedure called percentage of non-overlapping data (PND) (61). PND is used in single-case analysis studies as one of the alternatives to visual trend analysis (37). For this technique, the percentage of data points during the treatment period which exceeds the best performance data within the baseline phase is used to index the effectiveness of an intervention. Here, effect size is based on the percentage of non-overlapping data between baseline and treatment phases of the single-case intervention trial: highly effective ≥ 90%, moderately effective 70-90%, minimally effective 50-70%, ineffective < 50% (3; 61).

## Results

In order to compare the patients’ performance with the performance of neurologically intact participants, the results of the same variables from the healthy non-gamer participants (18 subjects, 9 male, mean age: 23.94± 3.56), conducted in our laboratory, is also shown in the graphs.

### Game performance

Patient 1 finished all 17 missions in the single player mode of the game and started the Special Ops mode of the game. Patients 2 and 3 completed 3 and 15 missions respectively.

### Self-paced saccades

**Figure 2:**
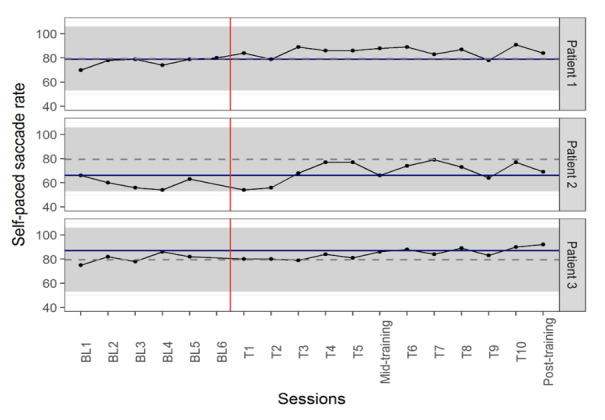
Self-paced saccade rate changes during baseline and training sessions (The number of self-paced saccades during 30 seconds was counted). Solid blue line represents the highest baseline value for each patient. Grey dashed lines and grey backgrounds show the mean ± standard deviation in healthy non-gamers participants. The red vertical line separates the baseline and training periods. BL = baseline and T= training day.

PND analysis showed an effect size of 80% in patient 1, as 8 out of 10 training values were higher than highest baseline value (80 on day 6). Therefore, intervention was moderately effective in this patient (PND=80%). For patient 2, 7 out of 10 training values were higher than the highest baseline value, showing a moderately effective result (PND=70%). Patient 3 had 3 data points higher than the highest baseline value, thus intervention was ineffective in this patient (PND=30%) (Figure 2).

### Abstract visual search

To investigate the accuracy of responses in visual search task, eye movements were manually checked to ensure that when patient pressed the button, the last fixation before ending the trial was on the location of the red letter “b”.

### Search time

**Figure 3:**
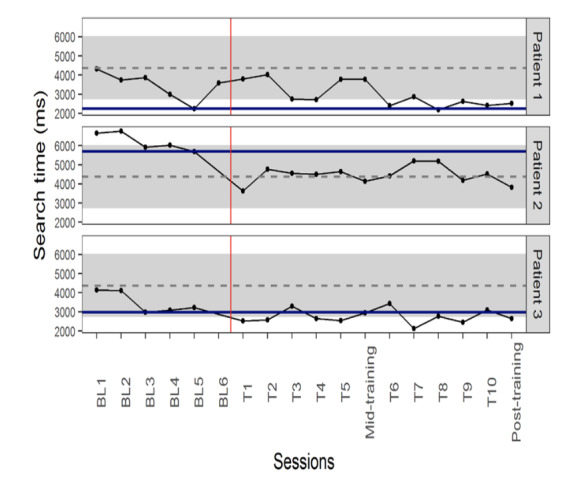
Manual search time (ms) changes during the baseline and training sessions, solid blue line represents the shortest baseline value for each patient. Grey dashed lines and grey backgrounds show the mean ± standard deviation search time in healthy non-gamer participants. *The red vertical line separates baseline and training periods. BL= baseline, T=training day.*

As shown in Figure 3, PND for patient 1 was 10%, i.e. 1 point out of 10 in the training period was shorter than the fastest search time in baseline, which is consistent with an ineffective training effect. In contrast, data for patient 2 showed a highly effective result (PND = 100%) with all training search times less than the shortest baseline value (5697 ms). The post-training time also remained less than the shortest baseline value. A minimally-moderately effective change was evident for patient 3 with 7 of 10 (70%) training search times shorter than the shortest baseline search time (2986 ms). The post training time remained less than the shortest baseline value.

### Fixation duration (FD)

**Figure 4:**
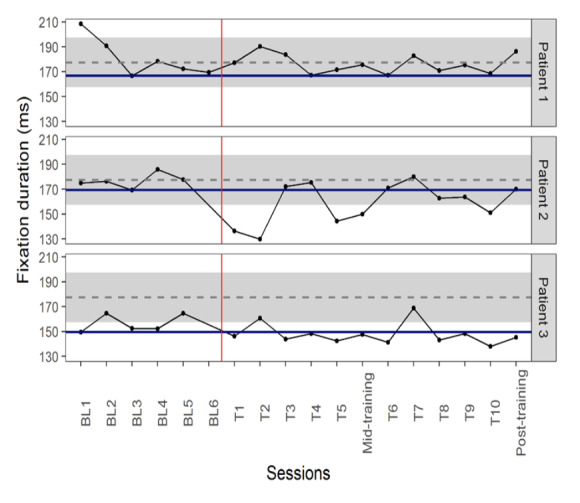
Fixation duration (FD: ms) changes during the baseline and training sessions. Solid blue line represents the shortest baseline value for each patient. Grey dashed lines and grey backgrounds show the mean ± standard deviations of FD in healthy non-gamer participants. The red vertical line separates baseline and training periods. BL= baseline, T=training

No training effect was evident for patient 1. FD was lowest on day three of baseline testing (166.6 ms) and there was no data point in the training period less than this value (PND= 0%). It is noteworthy that all but one of the baseline FD values and all the training FD values for patient 1 were within one standard deviation of the mean of healthy participants. A minimally effective training result (PND= 60%) was evident for patient 2 (6/10 training points had shorter FD than the lowest baseline data point of 169.2 ms); however, this dropped to baseline value in the post-training session. Patient 3 showed a moderately effective result (PND =80%) with 8 out of 10 training points having a shorter latency than the shortest baseline point (149.6 ms). Her baseline performance was even shorter than the normal range (Figure 4).

## Discussion

This exploratory experiment investigated the influence of action video game training on visual attention in three adults with severe TBI, as tested by performance on the self-paced saccade and visual search task.

Self-paced saccade was within one standard deviation of normal range in all three participants before the intervention. We observed a consistent increase in the rate of self-paced saccades in patients 1 and 2, which indicated moderately effective intervention in these patients. Search time was almost within normal range in baseline in all subjects, except patient 2 that started the task slowly but became faster at the end of the training. Patients 2 and 3 showed highly to moderately effective intervention in their manual search times, with faster reaction times that remained stable post training. Fixation duration was within normal limits in patients 1 and 2, but not in patient 3 (shorter than normal limits). After the training, there was no change in fixation duration in patients 1; however, patients 2 and 3 showed a minimal and moderately effective intervention in duration of fixation. These results might suggest a few modifications of visual attention after action video game training. Below, these results will be discussed for each patient separately.

### Patient 1

Patient 1 showed a consistent increase in self-paced saccade rate, which suggested a moderate effect of the intervention. However, there was no evidence of intervention on search time and FD in abstract visual search. Self-paced saccades involve a purely endogenous initiation of a saccade without any reflexive trigger. They require a quick volitional engagement and disengagement of attention between two constantly present stimuli (1). The FEF and DLPFC have been shown to be involved in generation of self-paced saccades (49). Kuhn et al. (45) found a positive correlation between volume of grey matter in the left FEF and left DLPFC and frequency of video gaming. The average video gaming experience in their study was 12.6 hours per week. While attributing the observed change in our patient entirely to the 10 hours of game training might not be possible, there is evidence that synaptic reorganization including dendritic arborization and axonal sprouting can occur within minutes (14) to days and weeks (15). Brain mapping studies have found that extensive experience with certain skills can enlarge brain structures involved in that activity. Modifications in brain structures in tasks such as keyboard typing (10), musical skills (18) and taxi driving (51) have been observed.

In terms of search time, patient’s fastest performance occurred on day 5 of baseline, where he showed a very fast reaction time, even faster than the normal range; however, he returned to normal range limit on day 6 of baseline. As PND, considers the fastest baseline performance to investigate the intervention effect, performance on day 5 might have acted as an outlier and have covered any effect of the training,

### Patient 2

This patient showed a moderate effect of game-training on the rate of self-paced saccades. As discussed for patient 1, this increase might have occurred as a result of plastic changes in FEF and DLPFC grey matter (45). Her performance in the abstract search task showed a high intervention effect in search time and minimum intervention effect in FD. She started the task with slower reaction times than healthy participants and became faster, although still in the normal range, at the end of the training. Faster reaction times following game playing have been one of the most consistent findings reported in the video game studies (17; H^33^). Playing action video games requires fast processing of sensory information and making very fast decisions to act instantly in order to avoid penalties or losing lives. This possibly justifies improvement in manual reaction time after action game training.

Patient 2 showed a minimal effect on FD after the training, but her performance was not constant. She started in the range of healthy participants and then became faster during training to reach durations even shorter than normal participants in four days of the training. However, in the post-training session, her FD returned to the baseline range. Given that it was a minimal effect and it was not maintained at the end of the training, the changes that were observed in FD are unlikely to have occurred as a result of training.

### Patient 3

This patient did not show any effect of intervention in the self-paced saccade task. However, she showed a minimal to moderate effect of intervention in search time and a moderate effect in FD in the abstract visual search. Her search time at baseline was already in the lower range of healthy participants. However, it became even shorter during the training; the amount of change was small, possibly because there was little room to improve. This result raises the question of whether or not there was any evidence of a speed-accuracy trade-off. Investigating her performance on abstract search task, we found almost 100% accuracy on all days; therefore, her very fast performance is unlikely to be due to sacrificing accuracy for speed. Interestingly, her FD at baseline was shorter than healthy participants and it became shorter during the training; however, the amount of change was small, i.e. about 10 ms shorter FD than baseline on the last day of the training. The small amount of change might be attributed to her shorter than normal duration of fixation in baseline, which affords little or no room for improvement. The consistently fast performance of this patient in search time and FD might arise from the possibility that she was exceptionally fast even before the brain injury. However, it might also have been as a result of the injury. Impairment or malfunction within specific centers of the collicular fixation neurons can bring shorter than normal fixation duration in brain injured patients or even healthy people with the ability to make more than 30% express saccades in a standard overlap task (7). Shorter FD along with faster search time after training might be attributed to the game intervention. There is evidence that search time is correlated with the number of fixations and duration of fixations (31; 73).

## General Discussion

Our results suggest different influences of action video gaming on different metrics measured in each TBI patient. This is not unexpected given the differences in time since injury, various sites of the injury, and the time of intervention, indicating the advantage of a SCED. In SCED, each subject serves as both control and experimental condition by collecting multiple baseline data and a causal or functional relationship between dependent and independent variables are investigated (32).

Overall, patient 2 appeared to benefit more than the other patients. She had a severe TBI with LOC and PTA of 2 months. While she struggled with learning the game at the start of the training, her reactions to game events improved during training such that she could complete 3 levels by the end of the intervention.

In this exploratory study, we employed eye movement recording to investigate progress in rehabilitation in TBI patients. Self-paced saccade has been recognized as a diagnostic marker of TBI (34; 69); however, its application in TBI rehabilitation has not been assessed previously. We examined it for the first time. Two of our three cases showed improvement in the self-paced saccade task after the training. This pilot data might justify its potential usage in rehabilitation studies. However, randomized control trial (RCT) studies are required to demonstrate the efficacy of this test as an objective measure for monitoring of TBI patients in rehabilitation process.

Overall, one might ask how brain injured participants were able to play a fast-paced game? This is a valid concern, as finding an interested participant who was also able to work with a computer was particularly difficult. Many TBI patients (moderate and severe) suffer from motor disabilities involving their hand and arm movements. Moreover, getting them to play a shooting game that even many healthy non-gamer adults might refuse is a challenge. The graphical user interface for the commercial shooter games might be too demanding for patients with impairments in information processing, visual and motor abilities. There is no way to adjust the level of difficulty according to each patient’s reaction times and information processing abilities in a commercial video game. A custom-made video game might compensate for the limitations of a commercial video game including control of the difficulty level in a trial by trial manner as well as incorporating some specific features in a theory-driven manner. Montani, De Grazia, and Zorzi (54) designed an adaptive video game for training attention and executive functions in populations such as patients with TBI. Their video game “Labyrinth” contains 3 different tasks aimed at training task switching, dual tasking and planning, cognitive functions that are often impaired after a TBI. They validated the game in a group of healthy participants by observing the training effect. Training duration was 40 minutes daily for 14 days. One of the most important features of the game, they claim, is the continuous process of calibration of difficulty level of the game according to the gamers’ performance. This feature could control the variability of the performance in each patient. Another concern with using an action shooter game is the violence theme which might be a contraindication in TBI patients. The long term influence of playing violent games on promoting aggression and emotional changes on normal adults showed no significant changes obtained on a large battery of tests (44); however, this might not be applicable in TBI patients. To be on the safe side, a custom-made video game with no violence theme is recommended.

The question of whether or not the amount of training was enough to achieve optimal results is also valid. Vakili and Langdon (70) applied a first-person shooter game to improve attentional abilities, executive functions and quality of life in 16 TBI patients. They showed improvement in an AB task after 10 hours of training. Notably, ten hours of game playing has been shown to have positive effects on search time (77), useful field of view and attentional blink in healthy participants (19; 21, 22, 23). However, investigating a longer period of game training in TBI patients is worth examining, as rehabilitation of cognitive domain might require more time in patients with more severe injury.

### Limitations

Despite finding some positive changes in eye movements and search time measures, the durability and transferability of these changes remains unknown. Future studies should assess changes over a longer period of time and also include measures which more directly examine real life skills, as the main aim of rehabilitation is to improve daily life activities. This study also lacked in using control measures related to untrained behavior in the experiment which could control for the influence of spontaneous recovery, practice effects and general stimulation. Although, several baseline testing possibly control for any practice effect (32), but having an unrelated measure could have covered for this flaw. At last, one limitation of the PND is that the effect size is measured based on only one data point in the baseline which makes it vulnerable to an outlier if it reaches to floor or ceiling of the score range, covering any influence of training. This might be true for search time in patient 1.

### Conclusion

This exploratory study investigated the use of commercial action video games to improve attentional abilities in patients with TBI measured with eye movement variables. Overall, some improvements were observed. These results may provide a baseline to design RCT studies using custom-made video games to validate the usefulness of this novel method of cognitive training and further investigate eye movements’ measures especially self-paced saccades as a marker of rehabilitation process.

### Ethics and Conflict of Interest

The authors declare that the contents of the article are in agreement with the ethics described in http://biblio.unibe.ch/portale/elibrary/BOP/jemr/ethics.html and that there is no conflict of interest regarding the publication of this paper.

## References

[b1] Abel, L. A., & Douglas, J. (2007). Effects of age on latency and error generation in internally mediated saccades. Neurobiology of Aging, 28(4), 627–637. 10.1016/j.neurobiolaging.2006.02.003 1558-1497 16540205

[b2] Achtman, R. L., Green, C. S., & Bavelier, D. (2008). Video games as a tool to train visual skills. Restorative Neurology and Neuroscience, 26(4-5), 435–446.0922-6028 18997318 PMC2884279

[b3] Alresheed, F., Hott, B. L., & Bano, C. (2013). Single Subject Research: A Synthesis of Analytic Methods. The Journal of Special Education Apprenticeship, 2(1), n1.2167-3454

[b4] Anguera, J. A., Boccanfuso, J., Rintoul, J. L., Al-Hashimi, O., Faraji, F., Janowich, J., Kong, E., Larraburo, Y., Rolle, C., Johnston, E., & Gazzaley, A. (2013). Video game training enhances cognitive control in older adults. Nature, 501(7465), 97–101. 10.1038/nature12486 1476-4687 24005416 PMC3983066

[b5] Azizi, E., Abel, L. A., & Stainer, M. J. (2017). The influence of action video game playing on eye movement behaviour during visual search in abstract, in-game and natural scenes. Attention, Perception & Psychophysics, 79(2), 484–497. 10.3758/s13414-016-1256-7 1943-393X 27981521

[b6] Belchior, P., Marsiske, M., Sisco, S. M., Yam, A., Bavelier, D., Ball, K., & Mann, W. C. (2013). Video game training to improve selective visual attention in older adults. Computers in Human Behavior, 29(4), 1318–1324. 10.1016/j.chb.2013.01.034 0747-5632 24003265 PMC3758751

[b7] Biscaldi, M., Fischer, B., & Stuhr, V. (1996). Human express saccade makers are impaired at suppressing visually evoked saccades. Journal of Neurophysiology, 76(1), 199–214. 10.1152/jn.1996.76.1.199 0022-3077 8836219

[b8] Bohnen, N., Jolles, J., & Twijnstra, A. (1992). Neuropsychological deficits in patients with persistent symptoms six months after mild head injury. Neurosurgery, 30(5), 692–695.0148-396X 1584379

[b9] Brouwer, W., Verzendaal, M., van der Naalt, J., Smit, J., & van Zomeren, E. (2001). Divided attention years after severe closed head injury: The effect of dependencies between the subtasks. Brain and Cognition, 46(1-2), 54–56. 10.1016/S0278-2626(01)80033-6 0278-2626 11527363

[b10] Cannonieri, G. C., Bonilha, L., Fernandes, P. T., Cendes, F., & Li, L. M. (2007). Practice and perfect: Length of training and structural brain changes in experienced typists. Neuroreport, 18(10), 1063–1066. 10.1097/WNR.0b013e3281a030e5 0959-4965 17558297

[b11] Cicerone, K. D., Dahlberg, C., Malec, J. F., Langenbahn, D. M., Felicetti, T., Kneipp, S., Ellmo, W., Kalmar, K., Giacino, J. T., Harley, J. P., Laatsch, L., Morse, P. A., & Catanese, J. (2005). Evidence-based cognitive rehabilitation: Updated review of the literature from 1998 through 2002. Archives of Physical Medicine and Rehabilitation, 86(8), 1681–1692. 10.1016/j.apmr.2005.03.024 0003-9993 16084827

[b12] Cicerone, K. D., Langenbahn, D. M., Braden, C., Malec, J. F., Kalmar, K., Fraas, M., Felicetti, T., Laatsch, L., Harley, J. P., Bergquist, T., Azulay, J., Cantor, J., & Ashman, T. (2011). Evidence-based cognitive rehabilitation: Updated review of the literature from 2003 through 2008. Archives of Physical Medicine and Rehabilitation, 92(4), 519–530. 10.1016/j.apmr.2010.11.015 1532-821X 21440699

[b13] Corrigan, J. D., & Bogner, J. (2007). Initial reliability and validity of the Ohio State University TBI identification method. The Journal of Head Trauma Rehabilitation, 22(6), 318–329. 10.1097/01.HTR.0000300227.67748.77 0885-9701 18025964

[b14] Dinse, H. R., Recanzone, G. H., & Merzenich, M. M. (1993). Alterations in correlated activity parallel ICMS-induced representational plasticity. Neuroreport, 5(2), 173–176. 10.1097/00001756-199311180-00020 0959-4965 8111006

[b15] Donoghue, J. P. (1995). Plasticity of adult sensorimotor representations. Current Opinion in Neurobiology, 5(6), 749–754. 10.1016/0959-4388(95)80102-2 0959-4388 8805413

[b16] Dye, M. W. G., & Bavelier, D. (2010). Differential development of visual attention skills in school-age children. Vision Research, 50(4), 452–459. 10.1016/j.visres.2009.10.010 1878-5646 19836409 PMC2824025

[b17] Dye, M. W. G., Green, C. S., & Bavelier, D. (2009b). Increasing speed of processing with action video games. Current Directions in Psychological Science, 18(6), 321–326. 10.1111/j.1467-8721.2009.01660.x 0963-7214 20485453 PMC2871325

[b18] Elbert, T., Pantev, C., Wienbruch, C., Rockstroh, B., & Taub, E. (1995). Increased cortical representation of the fingers of the left hand in string players. Science, 270(5234), 305–307. 10.1126/science.270.5234.305 0036-8075 7569982

[b19] Feng, J., Spence, I., & Pratt, J. (2007). Playing an action video game reduces gender differences in spatial cognition. Psychological Science, 18(10), 850–855. 10.1111/j.1467-9280.2007.01990.x 0956-7976 17894600

[b20] Franceschini, S., Gori, S., Ruffino, M., Viola, S., Molteni, M., & Facoetti, A. (2013). Action video games make dyslexic children read better. Current Biology, 23(6), 462–466. 10.1016/j.cub.2013.01.044 1879-0445 23453956

[b21] Green, C. S., & Bavelier, D. (2003). Action video game modifies visual selective attention. Nature, 423(6939), 534–537. 10.1038/nature01647 0028-0836 12774121

[b22] Green, C. S., & Bavelier, D. (2006a). Effect of action video games on the spatial distribution of visuospatial attention. Journal of Experimental Psychology. Human Perception and Performance, 32(6), 1465–1478. 10.1037/0096-1523.32.6.1465 0096-1523 17154785 PMC2896828

[b23] Green, C. S., & Bavelier, D. (2006b). Enumeration versus multiple object tracking: The case of action video game players. Cognition, 101(1), 217–245. 10.1016/j.cognition.2005.10.004 0010-0277 16359652 PMC2896820

[b24] Green, C. S., & Bavelier, D. (2008). Exercising your brain: A review of human brain plasticity and training-induced learning. Psychology and Aging, 23(4), 692–701. 10.1037/a0014345 0882-7974 19140641 PMC2896818

[b25] Heitger, M. H., Anderson, T. J., Jones, R. D., Dalrymple-Alford, J. C., Frampton, C. M., & Ardagh, M. W. (2004). Eye movement and visuomotor arm movement deficits following mild closed head injury. Brain, 127(Pt 3), 575–590.0006-8950 14736751 10.1093/brain/awh066

[b26] Heitger, M. H., Jones, R. D., Macleod, A. D., Snell, D. L., Frampton, C. M., & Anderson, T. J. (2009). Impaired eye movements in post-concussion syndrome indicate suboptimal brain function beyond the influence of depression, malingering or intellectual ability. Brain, 132(Pt 10), 2850–2870. 10.1093/brain/awp181 1460-2156 19617197

[b27] Heitger, M. H., Macaskill, M. R., Jones, R. D., & Anderson, T. J. (2005). The impact of mild closed head injury on involuntary saccadic adaptation: Evidence for the preservation of implicit motor learning. Brain Injury : [BI], 19(2), 109–117. 10.1080/02699050410001720095 0269-9052 15841755

[b28] Henderson, J. M., Williams, C. C., Castelhano, M. S., & Falk, R. J. (2003). Eye movements and picture processing during recognition. Perception & Psychophysics, 65(5), 725–734. 10.3758/BF03194809 0031-5117 12956580

[b29] Hoffman, J. E., & Subramaniam, B. (1995). The role of visual attention in saccadic eye movements. Perception & Psychophysics, 57(6), 787–795. 10.3758/BF03206794 0031-5117 7651803

[b30] Hogendoorn, H. (2016). Voluntary saccadic eye movements ride the attentional rhythm. Journal of Cognitive Neuroscience, 28(10), 1625–1635. 10.1162/jocn_a_00986 1530-8898 27243615

[b31] Hooge, I. T. C., & Erkelens, C. J. (1999). Peripheral vision and oculomotor control during visual search. Vision Research, 39(8), 1567–1575. 10.1016/S0042-6989(98)00213-2 0042-6989 10343822

[b32] Horner, R. H., Carr, E. G., Halle, J., McGee, G., Odom, S., & Wolery, M. (2005). The use of single-subject research to identify evidence-based practice in special education. Exceptional Children, 71(2), 165–179. 10.1177/001440290507100203 0014-4029

[b33] Hubert-Wallander, B., Green, C. S., Sugarman, M., & Bavelier, D. (2011). Changes in search rate but not in the dynamics of exogenous attention in action videogame players. Attention, Perception & Psychophysics, 73(8), 2399–2412. 10.3758/s13414-011-0194-7 1943-393X 21901575

[b34] Hunfalvay, M., Roberts, C.-M., Murray, N., Tyagi, A., Kelly, H., & Bolte, T. (2019). Horizontal and vertical self-paced saccades as a diagnostic marker of traumatic brain injury. Concussion, 4(1), CNC60. 10.2217/cnc-2019-0001 2056-3299 31467684 PMC6714073

[b35] James, W. (1890). The perception of reality. Principles of psychology, 2, 283-324.

[b36] Jeon, S. T., Maurer, D., & Lewis, T. L. (2012). The effect of video game training on the vision of adults with bilateral deprivation amblyopia. Seeing and Perceiving, 25(5), 493–520. 10.1163/18784763-00002391 1878-4755 23193607

[b37] Kazdin, A. E., & Tuma, A. H. (1982). Single-case research designs.

[b38] Koepp, M. J., Gunn, R. N., Lawrence, A. D., Cunningham, V. J., Dagher, A., Jones, T., Brooks, D. J., Bench, C. J., & Grasby, P. M. (1998). Evidence for striatal dopamine release during a video game. Nature, 393(6682), 266–268. 10.1038/30498 0028-0836 9607763

[b39] Kowler, E., Anderson, E., Dosher, B., & Blaser, E. (1995). The role of attention in the programming of saccades. Vision Research, 35(13), 1897–1916. 10.1016/0042-6989(94)00279-U 0042-6989 7660596

[b40] Krasny-Pacini, A., & Evans, J. (2018). Single-case experimental designs to assess intervention effectiveness in rehabilitation: A practical guide. Annals of Physical and Rehabilitation Medicine, 61(3), 164–179. 10.1016/j.rehab.2017.12.002 1877-0665 29253607

[b41] Kratochwill, T. R., & Levin, J. R. (2015). Single-Case Research Design and Analysis (Psychology Revivals): New Directions for Psychology and Education. Routledge. 10.4324/9781315725994

[b42] Kraus, M. F., Little, D. M., Donnell, A. J., Reilly, J. L., Simonian, N., & Sweeney, J. A. (2007). Oculomotor function in chronic traumatic brain injury. Cognitive and Behavioral Neurology, 20(3), 170–178. 10.1097/WNN.0b013e318142badb 1543-3633 17846516

[b43] Kroll, A., Mak, M., & Samochowiec, J. (2016). Learning to search. The importance of eye movements in the decrease of response times during a visual choice reaction time task. Journal of Eye Movement Research, 9(5). 10.16910/jemr.9.5.5 1995-8692

[b44] Kühn, S., Kugler, D. T., Schmalen, K., Weichenberger, M., Witt, C., & Gallinat, J. (2019). Does playing violent video games cause aggression? A longitudinal intervention study. Molecular Psychiatry, 24(8), 1220–1234. 10.1038/s41380-018-0031-7 1476-5578 29535447 PMC6756088

[b45] Kühn, S., Lorenz, R., Banaschewski, T., Barker, G. J., Büchel, C., Conrod, P. J., Flor, H., Garavan, H., Ittermann, B., Loth, E., Mann, K., Nees, F., Artiges, E., Paus, T., Rietschel, M., Smolka, M. N., Ströhle, A., Walaszek, B., Schumann, G., . . . Gallinat, J., & the IMAGEN Consortium. (2014). Positive association of video game playing with left frontal cortical thickness in adolescents. PLoS One, 9(3), e91506. 10.1371/journal.pone.0091506 1932-6203 24633348 PMC3954649

[b46] Larose, S., Gagnon, S., Ferland, C., & Pépin, M. (1989). Psychology of computers: XIV. Cognitive rehabilitation through computer games. Perceptual and Motor Skills, 69(3 Pt 1), 851–858. 10.1177/00315125890693-126 0031-5125 2608401

[b47] Levin, H. S., Williams, D. H., Eisenberg, H. M., High, W. M., Jr., & Guinto, F. C., Jr. (1992). Serial MRI and neurobehavioural findings after mild to moderate closed head injury. Journal of Neurology, Neurosurgery, and Psychiatry, 55(4), 255–262. 10.1136/jnnp.55.4.255 0022-3050 1583509 PMC489036

[b48] Li, R. W., Ngo, C., Nguyen, J., & Levi, D. M. (2011). Video-game play induces plasticity in the visual system of adults with amblyopia. PLoS Biology, 9(8), e1001135. 10.1371/journal.pbio.1001135 1545-7885 21912514 PMC3166159

[b49] Lobel, E., Kahane, P., Leonards, U., Grosbras, M., Lehéricy, S., Le Bihan, D., & Berthoz, A. (2001). Localization of human frontal eye fields: Anatomical and functional findings of functional magnetic resonance imaging and intracerebral electrical stimulation. Journal of Neurosurgery, 95(5), 804–815. 10.3171/jns.2001.95.5.0804 0022-3085 11702871

[b50] Mack, D. J., & Ilg, U. J. (2014). The effects of video game play on the characteristics of saccadic eye movements. Vision Research, 102, 26–32. 10.1016/j.visres.2014.07.010 1878-5646 25091459

[b51] Maguire, E. A., Gadian, D. G., Johnsrude, I. S., Good, C. D., Ashburner, J., Frackowiak, R. S., & Frith, C. D. (2000). Navigation-related structural change in the hippocampi of taxi drivers. Proceedings of the National Academy of Sciences of the United States of America, 97(8), 4398–4403. 10.1073/pnas.070039597 0027-8424 10716738 PMC18253

[b52] Mateer, C., Sohlberg, M. M., & Youngman, P. K. (1990). The management of acquired attention and memory deficits. Cognitive Rehabilitation in Perspective, 68–95.

[b53] McDowell, J. E., Dyckman, K. A., Austin, B. P., & Clementz, B. A. (2008). Neurophysiology and neuroanatomy of reflexive and volitional saccades: Evidence from studies of humans. Brain and Cognition, 68(3), 255–270. 10.1016/j.bandc.2008.08.016 1090-2147 18835656 PMC2614688

[b54] Montani, V., De Grazia, M. D. F., & Zorzi, M. (2014). A new adaptive videogame for training attention and executive functions: design principles and initial validation. 10.3389/fpsyg.2014.00409 PMC4026745 24860529

[b55] Mulhall, L. E., Williams, I. M., & Abel, L. A. (1999). Bedside tests of saccades after head injury. Journal of Neuro-Ophthalmology, 19(3), 160–165. 10.1097/00041327-199909000-00002 1070-8022 10494943

[b56] Oei, A. C., & Patterson, M. D. (2013). Enhancing cognition with video games: A multiple game training study. PLoS One, 8(3), e58546. 10.1371/journal.pone.0058546 1932-6203 23516504 PMC3596277

[b57] Petit, L., Orssaud, C., Tzourio, N., Crivello, F., Berthoz, A., & Mazoyer, B. (1996). Functional anatomy of a prelearned sequence of horizontal saccades in humans. The Journal of Neuroscience : The Official Journal of the Society for Neuroscience, 16(11), 3714–3726. 10.1523/JNEUROSCI.16-11-03714.1996 0270-6474 8642414 PMC6578842

[b58] Ponsford, J., & Kinsella, G. (1992). Attentional deficits following closed-head injury. Journal of Clinical and Experimental Neuropsychology, 14(5), 822–838. 10.1080/01688639208402865 1380-3395 1474148

[b59] Robertson, I. H., Ward, T., Ridgeway, V., & Nimmo-Smith, I. (1994). The test of everyday attention (TEA). Thames Valley Test Company. 10.1017/s1355617700001697 9375156

[b60] Romeiser-Logan, L., Slaughter, R., & Hickman, R. (2017). Single-subject research designs in pediatric rehabilitation: A valuable step towards knowledge translation. Developmental Medicine and Child Neurology, 59(6), 574–580. 10.1111/dmcn.13405 1469-8749 28224606

[b61] Scruggs, T. E., Mastropieri, M. A., & Casto, G. (1987). The quantitative synthesis of single-subject research methodology and validation. Remedial and Special Education, 8(2), 24–33. 10.1177/074193258700800206 0741-9325

[b62] Segal, K. R., & Dietz, W. H. (1991). Physiologic responses to playing a video game. American Journal of Diseases of Children, 145(9), 1034–1036.0002-922X 1877563 10.1001/archpedi.1991.02160090086030

[b63] Shapiro, K. L., Raymond, J. E., & Arnell, K. M. (1997). The attentional blink. Trends in Cognitive Sciences, 1(8), 291–296. 10.1016/S1364-6613(97)01094-2 1364-6613 21223931

[b64] Smith, D. H., Meaney, D. F., & Shull, W. H. (2003). Diffuse axonal injury in head trauma. The Journal of Head Trauma Rehabilitation, 18(4), 307–316. 10.1097/00001199-200307000-00003 0885-9701 16222127

[b65] Sohlberg, M. M., Avery, J., Kennedy, M., Ylvisaker, M., Coelho, C., Turkstra, L., & Yorkston, K. (2003). Practice guidelines for direct attention training. Journal of Medical Speech-Language Pathology, 11(3), xix–xix.1065-1438

[b66] Sparks, D. L. (2002). The brainstem control of saccadic eye movements. Nature Reviews. Neuroscience, 3(12), 952–964. 10.1038/nrn986 1471-003X 12461552

[b67] Spikman, J. M., Deelman, B. G., & van Zomeren, A. H. (2000). Executive functioning, attention and frontal lesions in patients with chronic CHI. Journal of Clinical and Experimental Neuropsychology, 22(3), 325–338. 10.1076/1380-3395(200006)22:3;1-V;FT325 1380-3395 10855041

[b68] Spikman, J. M., van Zomeren, A. H., & Deelman, B. G. (1996). Deficits of attention after closed-head injury: Slowness only? Journal of Clinical and Experimental Neuropsychology, 18(5), 755–767. 10.1080/01688639608408298 1380-3395 8941860

[b69] Taghdiri, F., Chung, J., Irwin, S., Multani, N., Tarazi, A., Ebraheem, A., Khodadadi, M., Goswami, R., Wennberg, R., Mikulis, D., Green, R., Davis, K., Tator, C., Eizenman, M., & Tartaglia, M. C. (2018). Decreased number of self-paced saccades in post-concussion syndrome associated with higher symptom burden and reduced white matter integrity. Journal of Neurotrauma, 35(5), 719–729. 10.1089/neu.2017.5274 1557-9042 29239265

[b70] Vakili, A., & Langdon, R. (2016). Cognitive rehabilitation of attention deficits in traumatic brain injury using action video games: A controlled trial. Cogent Psychology, 3. 10.1080/23311908.2016.1143732 2331-1908

[b71] Van der Stigchel, S., & Nijboer, T. C. (2011). The global effect: What determines where the eyes land? Journal of Eye Movement Research, 4(2).1995-8692

[b72] Vassallo, S., & Douglas, J. (2021). Visual scanpath training to emotional faces following severe traumatic brain injury: A single case design. Journal of Eye Movement Research, 14(4).1995-8692 34760060 10.16910/jemr.14.4.6 PMC8575428

[b73] Wienrich, C., Heße, U., & Müller-Plath, G. (2009). Eye movements and attention in visual feature search with graded target-distractor-similarity. Journal of Eye Movement Research, 3(1). 10.16910/jemr.3.1.4 1995-8692

[b74] Williams, I. M., Ponsford, J. L., Gibson, K. L., Mulhall, L. E., Curran, C. A., & Abel, L. A. (1997). Cerebral control of saccades and neuropsychological test results after head injury. Journal of Clinical Neuroscience, 4(2), 186–196. 10.1016/S0967-5868(97)90072-2 0967-5868 18638954

[b75] Withaar, F. K., & Brouwer, W. H. (2003). Divided attention after closed head injury. Zeitschrift für Neuropsychologie, 14(3), 203–211. 10.1024/1016-264X.14.3.203 1016-264X

[b76] Wolfe, J. M. (1994). Guided Search 2.0 A revised model of visual search. Psychonomic Bulletin & Review, 1(2), 202–238. 10.3758/BF03200774 1069-9384 24203471

[b77] Wu, S., & Spence, I. (2013). Playing shooter and driving videogames improves top-down guidance in visual search. Attention, Perception & Psychophysics, 75(4), 673–686. 10.3758/s13414-013-0440-2 1943-393X 23460295

